# Research utility of the National Violent Death Reporting System: a scoping review

**DOI:** 10.1186/s40621-019-0196-9

**Published:** 2019-05-20

**Authors:** Oybek Nazarov, Joseph Guan, Stanford Chihuri, Guohua Li

**Affiliations:** 1 Department of Anesthesiology, Columbia University Vagelos College of Physicians and Surgeons, New York, NY USA; 2 Rush Medical College, Chicago, IL USA; 3 Center for Injury Epidemiology and Prevention, Columbia University Irving Medical Center, 622 West 168th St, PH5-534, New York, NY 10032 USA; 4 Department of Epidemiology, Columbia University Mailman School of Public Health, New York, NY USA

**Keywords:** Epidemiology, Homicide, Injury, Knowledge domain, National Violent Death Reporting System, Scoping review, Suicide, Violence

## Abstract

**Background:**

To better understand and prevent suicide and homicide, the National Center for Injury Prevention and Control of the US Centers for Disease Control and Prevention launched the National Violent Death Reporting System (NVDRS) in six states in 2002. As of 2018, the NVDRS has been expanded to include all 50 states, the District of Columbia and Puerto Rico. The purpose of this review was to assess the research utility of the NVDRS based on studies indexed in major bibliographical databases.

**Methods:**

We performed a scoping review of published studies that were based on data from the NVDRS, identified by searching six electronic databases: PubMed, EMBASE, Google Scholar, OVID, Scopus, and Web of Science. We examined the time trend of annual NVDRS-based research output, generated a word cloud using the keywords listed in the publications, and mapped the knowledge domains covered by NVDRS-based studies.

**Results:**

Our review included a total of 150 studies published between 2005 and 2018. There was a marked increase in the annual number of NVDRS-based publications, with 120 (80.0%) of the 150 studies published between 2011 and 2018. Overall, 104 (69.3%) studies focused on suicide and 39 (26.0%) on homicide. Of the included studies, 100 (66.7%) were descriptive epidemiology, 31 (20.7%) were risk factor analyses, 9 (6.0%) were evaluations, 7 (4.7%) were trend analyses, and 4 (2.7%) were data quality assessments. Knowledge domain mapping identified two major clusters of studies, one on suicide and the other on homicide. The cluster on suicide was commonly linked to “circumstance,” “alcohol” and “substance abuse” and the cluster on homicide was commonly linked to “firearm,” “injury,” and “gang.” The two clusters were interlinked to overlapping networks of keywords, such as “firearm” and “mental health problem.”

**Conclusions:**

Research utility of the NVDRS has increased considerably in recent years. Studies based on data from the NVDRS are clustered in two knowledge domains – suicide and homicide. The vast potential of the NVDRS for violence research and prevention remains to be fully exploited.

## Background

In 2016, approximately 65,000 deaths were attributed to violence-related injuries in the United States (Fatal Injury Reports, 1981-2016). Of these violent deaths, about 70% resulted from suicide and 30% from homicide. Violent deaths account for about 30% of the total injury mortality in the United States and violence prevention presents a major public health challenge. Information about the circumstances surrounding violent deaths is crucial for developing effective intervention programs. In an effort to understand and prevent suicide and homicide, the National Center for Injury Prevention and Control of the Centers for Disease Control and Prevention (CDC) started the National Violent Death Reporting System (NVDRS) in 2002 to monitor violent deaths and their circumstances (Blair et al. 2016).

Prior to the establishment of the NVDRS, data sources such as the National Vital Statistics System and the Uniform Crime Reporting Statistics provided only limited information about the circumstances of violent deaths (Crosby et al. 2016). As such, there were calls for the creation of a national data system on violent deaths similar to the Fatality Analysis Reporting System developed by the National Highway Traffic Safety Administration (Mercy and Houk 1988; Teret et al. 1992; Barber et al. 2000; Hemenway et al. 2009). After several failed attempts at developing and enhancing violent injury surveillance systems, the CDC halted all activities related to violent injuries in 1996 (Blair et al. 2016; Rovner 1996). However, in 1998, a group of private foundations provided temporary funding to revive the initiative and in 1999, in response to an Institute of Medicine report calling for a national fatal injury system, a pilot program called the National Violent Injury Statistics System (NVISS) was established (Blair et al. 2016).

With growing support for the NVISS, in 2002 the Congress allocated $1.5 million to the CDC to establish the NVDRS (Paulozzi et al. 2004). It became the first multistate system to provide detailed information on the circumstances precipitating violent deaths by culling data from multiple sources (Crosby et al. 2016). In 2003, the surveillance system began data collection in six states: Maryland, Massachusetts, New Jersey, Oregon, South Carolina, and Virginia (Blair et al. 2016; McNally et al. 2016). Since then, the US government has provided an additional $20 million in annual funding to expand the NVDRS to collect data from all states (Barber et al. 2013). As of 2018, the NVDRS has been expanded to all 50 states, the District of Columbia, and Puerto Rico, pooling data from coroner and medical examiner reports, death certificates, law enforcement reports, and toxicology reports using standardized protocols.

Since the launch of the NVDRS, data generated from the system have been used to identify research needs of at-risk populations and develop targeted intervention programs to prevent violent deaths. For instance, the NVDRS data have been used to study homicide and suicide incidents involving older adults and young children, veterans, and law enforcement officers and to document the prevalence of putative risk factors such as alcohol abuse, substance abuse, and financial distress. One study found a significant increase in suicide related to the US housing crisis resulting in foreclosures (Fowler et al. 2015). Another study showed that veterans had a higher risk of suicide in comparison to non-veterans (Hemenway et al. 2009). The NVDRS has helped build alliances among key stakeholders, inform intervention programs, raise awareness, and catalyze new project developments (Horan and Mallonee 2003; Cambell et al. 2006). For instance, the Oregon Violent Death Reporting System staff collaborated with the Oregon Veteran Health Administration to establish prevention programs for veterans (Shen and Millet 2014).

Violent death is defined as a death resulting from either the intentional use of physical force or power against oneself or others (Blair et al. 2016). The NVDRS categorizes violent deaths into the following groups: suicide, homicide, unintentional firearm death, deaths of undetermined intent, deaths due to legal intervention (excluding executions) and deaths due to terrorism (excluding acts of war). Reportable cases are identified based on the codes of the International Classification of Diseases, Tenth Revision, or the manner of death assigned by the coroner, medical examiner or law enforcement (Blair et al. 2016). The NVDRS system is incident-based and records data on both the perpetrator and the victim. In the event of multiple-death homicide, it links all the deaths into one incident record. The NVDRS has been used extensively by researchers. The purpose of this scoping review is to provide a summary assessment of the research utility of the NVDRS through bibliographical analysis, including knowledge domain mapping and network visualization.

## Methods

This scoping review was an analysis of bibliographical data for NVDRS-based studies published in peer-reviewed journals. We followed the framework outlined by Arksey and O’Malley (2005).

### Eligibility criteria

In the first phase, only the titles and abstracts of potentially relevant studies were screened to match the minimum inclusion criteria. In case of titles for which abstracts were not available, full articles were obtained and reviewed. Publications that were letters, editorials, commentaries, or abstracts only were excluded. Also excluded were articles describing the NVDRS without analyzing the data. Studies eligible for inclusion in this review were those that were based solely or partially on the NVDRS data.

### Data sources and search criteria

The search query consisted of “National Violent Death Reporting System” and “NVDRS” and was conducted in six databases: PubMed, EMBASE, Google Scholar, OVID, Scopus, and Web of Science. Search criteria included all the articles published from 2002 to 2018 in English.

### Citation management

All citations were imported into the EndNote-PC software. Duplicate articles were manually excluded by sorting and reviewing individual article titles.

### Analytical tool

Bibliographical data from studies meeting the inclusion criteria were analyzed with the software VOSviewer (ver. 1.6.10) (Van Eck and Watman 2016) and the online word cloud generator (Word Clouds, 2019). VOSviewer is a literature knowledge visualization software based on the Visualization of Similarities (VOS) technology. It displays knowledge domain maps and clusters based on network data. VOSviewer is well suited for analyzing large-scale bibliographical data and for constructing complex networks and interactions. Using bibliographic and text data, co-occurrence network, citation network and coupling network can be constructed (Van Eck and Waltman 2010). In this review, we used VOSviewer to map knowledge domains and display the development process and structural relationships of the scientific knowledge covered in the studies. Keywords in the same cluster show the greater similarity with respect to the highest node keyword and positioned within the same cluster. The size of the text within each circle of the network visualization map corresponds to the weight/frequency of that keyword within and between the clusters. The software also provides a quantifiable framework through “Total Links Strength” and “Occurrences” analysis. Total links strength is calculated as: $\frac{\sum Links\ to\ other\ keywords}{Total\ links}$. The higher the total links strength, the higher the co-occurrence of given keywords. “Occurrences” are the total frequency of a keyword within the data (Van Eck and Waltman, 2016).

## Results

**Fig. 1:**
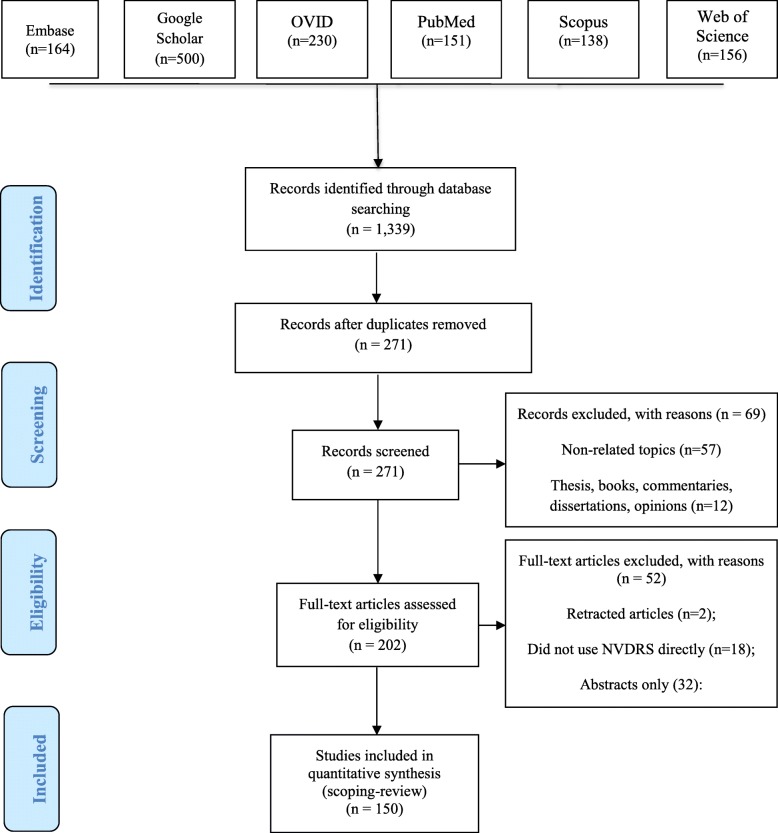
Flow diagram of identification, review, and selection of published studies based on data from the National Violent Death Reporting System (NVDRS), 2002–2018

The comprehensive search returned a total of 1339 potentially relevant articles. Of the 1339 articles, 151 were indexed in PubMed, 164 in Embase, 500 in Google Scholar, 230 in OVID, 138 in Scopus, and 156 in Web of Science. After removing duplicates, 271 articles were included for further review. Of these, 69 did not meet the eligibility criteria due to non-related topics or articles that were thesis, books, commentaries, dissertations, and opinions, leaving 202 articles that were reviewed in full-text. After excluding 52 full-text articles that contained retracted articles, did not use NVDRS or were abstracts only, 150 met the inclusion criteria and were included in the scoping review (Fig. 1 and Table 1). Of the 150 articles, 132 were indexed in PubMed and 147 in Google Scholar.

**Table 1:** Studies based on data from the National Violent Death Reporting System by year of publication, 2002–2018

Year of Publication ^a^	Citation
2005	Schecter WP, Klassen C, O’Connor P, Potts M, Ochitill H. Suicide, the unmet challenge of the trauma system. *Archives of Surgery*. 2005;140(9):902–4.
2006	Bennett MD, Jr., Hall J, Frazier L, Jr., Patel N, Barker L, Shaw K. Homicide of children aged 0–4 years, 2003–04: Results from the National Violent Death Reporting System. *Injury Prevention.* 2006;Suppl 2:ii39-43. Bossarte RM, Simon TR, Barker L. Characteristics of homicide followed by suicide incidents in multiple states, 2003–04. *Injury Prevention*. 2006;Suppl 2:ii33-8. Breiding MJ, Wiersema B. Variability of undetermined manner of death classification in the U.S. *Injury Prevention*. 2006;Suppl 2:ii49-54. Campbell R, Weis MA, Millet L, Powell V, Hull-Jilly D, Hackman H. From surveillance to action: early gains from the National Violent Death Reporting System. *Injury Prevention.* 2006;Suppl 2: ii6–9. Hempstead K. Manner of death and circumstances in fatal poisonings: evidence from New Jersey. *Injury Prevention.* 2006;Suppl 2 ii44-8. Karch DL, Barker L, Strine TW. Race/ethnicity, substance abuse, and mental illness among suicide victims in 13 U.S. states: 2004 data from the National Violent Death Reporting System. *Injury Prevention.* 2006;Suppl 2:ii22-7. Patel N, Webb K, White D, Barker L, Crosby A, DeBerry M. Homicides and suicides - National Violent Death Reporting System, United States, 2003–2004. *Morbidity and Mortality Weekly Report.* 2006;55(26):721–4. Powell V, Barber CW, Hedegaard H, Hempstead K, Hull-Jilly D, Shen X. Using NVDRS data for suicide prevention: promising practices in seven states. *Injury Prevention.* 2006;Suppl 2:ii28-32. Sanford C, Marshall SW, Martin SL, Coyne-Beasley T, Waller AE, Cook PJ. Deaths from violence in North Carolina, 2004: how deaths differ in females and males. *Injury Prevention.* 2006;Suppl 2:ii10-6. Weis M, Bradberry C, Carter LP, Ferguson J, Kozareva D. An exploration of human services system contacts prior to suicide in South Carolina: an expansion of the South Carolina Violent Death Reporting System. *Injury Prevention.* 2006;Suppl 2:ii17-21.
2007	Kegler SR. Applying the compound Poisson process model to the reporting of injury-related mortality rates. *Epidemiologic Perspectives and Innovations.* 2007; 16;4:1.
2008	Barber CW, Azrael D, Hemenway D, Olson LM, Nie C, Schaechter J. Suicides and suicide attempts following homicide: victim–suspect relationship, weapon type, and presence of antidepressants. *Homicide Studies*. 2008;12(3):285–97. Karch DL, Lubell KM, Friday J, Patel N, Williams DD, Centers for Disease Control and Prevention. Surveillance for violent deaths--National Violent Death Reporting System, 16 states, 2005. *Morbidity and Mortality Weekly Report Surveillance Summary*. 2008;57(3):1–45. Logan J, Hill HA, Black ML, Crosby AE, Karch DL, Barnes JD. Characteristics of perpetrators in homicide-followed-by-suicide incidents: National Violent Death Reporting System--17 U.S. states, 2003–2005. *American Journal of Epidemiology.* 2008;168(9):1056–64. Pamer C, Serpi T, Finkelstein J. Analysis of Maryland poisoning deaths using classification and regression tree (CART) analysis. AMIA annual symposium proceedings. *American Medical Informatics Association*. 2008.: 550–554. Shields RT, Ward BW. Comparison of the National Violent Death Reporting System and supplementary homicide report: potential benefits of integration. *Justice Research and Policy*. 2008;10(2):67–97.
2009	Fujiwara T, Barber C, Schaechter J, Hemenway D. Characteristics of infant homicides: findings from a U.S. multisite reporting system. *Pediatrics*. 2009;124(2):e210–7. Kaplan MS, McFarland BH, Huguet N. Characteristics of adult male and female firearm suicide decedents: findings from the National Violent Death Reporting System. *Injury Prevention*. 2009a;15(5):322–7. Kaplan MS, McFarland BH, Huguet N. Firearm suicide among veterans in the general population: findings from the National Violent Death Reporting System. *Journal of Trauma - Injury, Infection and Critical Care.* 2009b;67(3):503–7. Karch DL, Dahlberg LL, Patel N, Davis TW, Logan JE, Hill HA. Surveillance for violent deaths--National Violent Death Reporting System, 16 states, 2006. *Morbidity and Mortality Weekly Report Surveillance Summaries.* 2009;58(1):1–44. Logan JE, Karch DL, Crosby AE. Reducing “unknown” data in violent death surveillance: a study of death certificates, coroner/medical examiner and police reports from the National Violent Death Reporting System, 2003–2004. *Homicide Studies*. 2009;13(4):385–97. Wanta BT, Schlotthauer AE, Guse CE, Hargarten SW. The burden of suicide in Wisconsin’s older adult population. *Wisconsin Medical Journal.* 2009;108(2):87–93.
2010	Genovesi AL, Donaldson AE, Morrison BL, Olson LM. Different perspectives: a comparison of newspaper articles to medical examiner data in the reporting of violent deaths. *Accident; Analysis and Prevention.* 2010;42(2):445–51. Hemenway D, Barber C, Miller M. Unintentional firearm deaths: a comparison of other-inflicted and self-inflicted shootings. *Accident; Analysis and Prevention*. 2010;42(4):1184–8. Karch DL, Dahlberg LL, Patel N. Surveillance for violent deaths--National Violent Death Reporting System, 16 states, 2007. *Morbidity and Mortality Weekly Report Surveillance Summaries*. 2010;59(4):1–50. Klevens J, Leeb RT. Child maltreatment fatalities in children under 5: Findings from the National Violence Death Reporting System. *Child Abuse and Neglect.* 2010;34(4):262–6. Madkour AS, Martin SL, Halpern CT, Schoenbach VJ. Area disadvantage and intimate partner homicide: an ecological analysis of North Carolina counties, 2004–2006. *Violence and Victims.*2010;25(3):363. Ortega LA, Karch D. Precipitating circumstances of suicide among women of reproductive age in 16 U.S. states, 2003–2007. *Journal of Women’s Health* (2002). 2010;19(1):5–7. Styka AN, White DS, Zumwalt RE, Lathrop SL. Trends in adult suicides in New Mexico: utilizing data from the New Mexico violent Death Reporting System. *Journal of Forensic Sciences.* 2010;55(1):93–9.
2011	Barber C, Hemenway D. Too many or too few unintentional firearm deaths in official U.S. mortality data? Accident; *Analysis and Prevention*. 2011;43(3):724–31. Betz ME, Valley MA, Lowenstein SR, Hedegaard H, Thomas D, Stallones L. Elevated suicide rates at high altitude: sociodemographic and health issues may be to blame. *Suicide & Life-Threatening Behavior*. 2011;41(5):562–73. Hewes HA, Keenan HT, McDonnell WM, Dudley NC, Herman BE. Judicial outcomes of child abuse homicide. *Archives of Pediatrics and Adolescent Medicine.* 2011;165(10):918–21. Karch D. Sex differences in suicide incident characteristics and circumstances among older adults: surveillance data from the National Violent Death Reporting System-17 U.S. states, 2007–2009. *International Journal of Environmental Research and Public Health.* 2011;8(8):3479–95. Karch D, Nunn KC. Characteristics of elderly and other vulnerable adult victims of homicide by a caregiver: National Violent Death Reporting System—17 US states, 2003–2007. *Journal of Interpersonal Violence.* 2011;26(1):137–57. Karch DL, Logan J, Patel N. Surveillance for violent deaths - National Violent Death Reporting System, 16 states, 2008. *Morbidity and Mortality Weekly Report* *Surveillance Summaries*. 2011;60(10):1–49. Liem M, Barber C, Markwalder N, Killias M, Nieuwbeerta P. Homicide–suicide and other violent deaths: an international comparison. *Forensic Science International*. 2011;207(1–3):70–6. Logan J, Hall J, Karch D. Suicide categories by patterns of known risk factors: a latent class analysis. *Archives of General Psychiatry.* 2011;68(9):935–41. Smith SG, Basile KC, Karch D. Sexual homicide and sexual violence-associated homicide: findings from the National Violent Death Reporting System. *Homicide Studies.* 2011;15(2):132–53. Ward BW, Shields RT, Cramer BR. Integrating medical examiner and police report data: can this improve our knowledge of the social circumstances surrounding suicide? *Crisis*. 2011;32(3):160–8. Wasserman I, Stack S. Race, urban context, and Russian roulette: findings from the National Violent Death Reporting System, 2003–2006. *Suicide and Life-Threatening Behavior.* 2011;41(1):33–40.
2012	Bahraini NH, Gutierrez PM, Harwood JE, Huggins JA, Hedegaard H, Chase M. The Colorado Violent Death Reporting System: validity and utility of the veteran status variable. *Public Health Reports.* 2012;127(3):304–9. Clark DE, Qian J, Sihler KC, Hallagan LD, Betensky RA. The distribution of survival times after injury. *World Journal of Surgery.* 2012;36(7):1562–70. Dailey NJ, Norwood T, Moore ZS, Fleischauer AT, Proescholdbell S. Evaluation of the North Carolina Violent Death Reporting System, 2009. *North Carolina Medical Journal.* 2012;73(4):257–62. Gold KJ, Singh V, Marcus SM, Palladino CL. Mental health, substance use, and intimate partner problems among pregnant and postpartum suicide victims in the National Violent Death Reporting System. *General Hospital Psychiatry*. 2012;34(2):139–45. Huguet N, Kaplan MS, McFarland BH. Rates and correlates of undetermined deaths among African Americans: results from the National Violent Death Reporting System. *Suicide & Life-Threatening Behavior*. 2012;42(2):185–96. Kaplan MS, Huguet N, McFarland BH, Mandle JA. Factors associated with suicide by firearm among U.S. older adult men. *Psychology of Men & Masculinity.* 2012;13(1):65–74. Kaplan MS, McFarland BH, Huguet N, Conner K, Caetano R, Giesbrecht N. Acute alcohol intoxication and suicide: a gender-stratified analysis of the National Violent Death Reporting System. *Injury Prevention.* 2012;19(1):38–43. Kaplan MS, McFarland BH, Huguet N, Valenstein M. Suicide risk and precipitating circumstances among young, middle-aged, and older male veterans. *American Journal of Public Health.* 2012;102. Karch DL, Logan J, McDaniel D, Parks S, Patel N, Centers for Disease C. Surveillance for violent deaths--National Violent Death Reporting System, 16 states, 2009. *Morbidity and Mortality Weekly Report Surveillance Summaries.* 2012;61(6):1–43. Katz IR, McCarthy JF, Ignacio RV, Kemp J. Suicide among veterans in 16 states, 2005 to 2008: comparisons between utilizers and nonutilizers of Veterans Health Administration (VHA) services based on data from the National Death Index, the National Violent Death Reporting System, and VHA administrative records. *American Journal of Public Health.* 2012;102 Suppl 1:S105-10. Lord V. Factors influencing subjects’ observed level of suicide by cop intent. *Criminal Justice and Behavior.* 2012;39(12):1633–46. Manion T, Akinyemi A, Nooraddini I, Haile E. A comparison of suicide characteristics and precipitating circumstances by age group among Maryland residents: data from the Maryland Violent Death Reporting System, 2003–2009. *Suicidology Online.* 2012;3:131–7. Palladino CL, Singh V, Campbell J, Flynn H, Gold KJ. Homicide and suicide during the perinatal period: findings from the National Violent Death Reporting System. *Obstetric Anesthesia Digest.* 2012;32(4):217–8. Walsh S, Charnigo R. An ecological approach to preventing suicide using the National Violent Death Reporting System and county level health status data. *Suicidology Online.* 2012;3:92-107.
2013	Beyer KMM, Layde PM, Hamberger LK, Laud PW. Characteristics of the residential neighborhood environment differentiate intimate partner femicide in urban versus rural settings. *Journal of Rural Health.* 2013;29(3):281–93. Caetano R, Kaplan MS, Huguet N, McFarland BH, Conner K, Giesbrecht N. Acute alcohol intoxication and suicide among United States ethnic/racial groups: findings from the National Violent Death Reporting System. *Alcoholism, Clinical and Experimental Research.* 2013b;37(5):839–46. Gold KJ, Sen A, Schwenk TL. Details on suicide among U.S. physicians: data from the National Violent Death Reporting System. *General Hospital Psychiatry.* 2013;35(1):45–9. Karch DL, Logan J, McDaniel DD, Floyd C, Vagi KJ. Precipitating circumstances of suicide among youth aged 10–17 years by sex: data from the National Violent Death Reporting System, 16 states, 2005–2008. *Journal of Adolescent Health*. 2013;53 Suppl 1:S51-3. Liu RT, Kraines MA, Puzia ME, Massing-Schaffer M, Kleiman EM. Sociodemographic predictors of suicide means in a population-based surveillance system: findings from the National Violent Death Reporting System. *Journal of Affective Disorders*. 2013;151(2):449–54. Logan JE, Walsh S, Patel Nk, Hall JE. Homicide-followed-by-suicide incidents involving child victims. *American Journal of Health Behavior.* 2013;37(4):531–42. Sheehan CM, Rogers RG, Williams IV GW, Boardman JD. Gender differences in the presence of drugs in violent deaths. *Addiction*. 2013;108(3):547–55. Stallones L, Doenges T, Dik BJ, Valley MA. Occupation and suicide: Colorado, 2004–2006. *American Journal of Industrial Medicine.* 2013;56(11):1290–5.
2014	Bozzay ML, Liu RT, Kleiman EM. Gender and age differences in suicide mortality in the context of violent death: findings from a multi-state population-based surveillance system. *Comprehensive Psychiatry.* 2014;55(5):1077–84. Clark DE, Doolittle PC, Winchell RJ, Betensky RA. The effect of hospital care on early survival after penetrating trauma. *Injury Epidemiology.* 2014;1(1):1–9. Conner KR, Huguet N, Caetano R, Giesbrecht N, McFarland BH, Nolte KB. Acute use of alcohol and methods of suicide in a U.S. national sample. *American Journal of Public Health.* 2014;104(1):171–8. Huber RS, Coon H, Kim N, Renshaw PF, Kondo DG. Altitude is a risk factor for completed suicide in bipolar disorder. *Medical Hypotheses.* 2014;82(3):377–81. Huguet N, Kaplan MS, McFarland BH. The effects of misclassification biases on veteran suicide rate estimates. *American Journal of Public Health.* 2014;104(1):151–5. Kaplan MS, Huguet N, McFarland BH, Caetano R, Conner KR, Giesbrecht N. Use of alcohol before suicide in the United States. *Annals of Epidemiology.* 2014;24(8):588–92.e1–2. Lord VB. Police responses in officer-involved violent deaths: comparison of suicide by cop and non-suicide by cop incidents. *Police Quarterly.* 2014;17(1):79–100. Niederkrotenthaler T, Logan JE, Karch DL, Crosby A. Characteristics of U.S. suicide decedents in 2005–2010 who had received mental health treatment. *Psychiatric Services.* 2014;65(3):387–90. Parks SE, Johnson LL, McDaniel DD, Gladden M. Surveillance for violent deaths - National Violent Death Reporting System, 16 states, 2010. *Morbidity and Mortality Weekly Report Surveillance Summaries.* 2014;63(1):1–33. Searles VB, Valley MA, Hedegaard H, Betz ME. Suicides in urban and rural counties in the United States, 2006–2008. *Crisis: The Journal of Crisis Intervention and Suicide Prevention.* 2014;35(1):18. Smith SG, Fowler KA, Niolon PH. Intimate partner homicide and corollary victims in 16 states: National Violent Death Reporting System, 2003–2009. A*merican Journal of Public Health.* 2014;104(3):461–6.
2015	Beyer KM, Layde PM, Hamberger LK, Laud PW. Does neighborhood environment differentiate intimate partner femicides from other femicides? *Violence Against Women.* 2015;21(1):49–64. Caetano R, Kaplan MS, Huguet N, Conner K, McFarland BH, Giesbrecht N. Precipitating circumstances of suicide and alcohol intoxication among U.S. ethnic groups. *Alcoholism, Clinical and Experimental Research.* 2015;39(8):1510–7. Cerel J, Moore M, Brown MM, van de Venne J, Brown SL. Who leaves suicide notes? A six-year population-based study. *Suicide and Life-Threatening Behavior*. 2015;45(3):326–34. Fan MD. Disarming the dangerous: preventing extraordinary and ordinary violence. *Indiana Law Journal*. 2015;90(1):151–78. Fowler KA, Gladden RM, Vagi KJ, Barnes J, Frazier L. Increase in suicides associated with home eviction and foreclosure during the U.S. housing crisis: findings from 16 National Violent Death Reporting System states, 2005–2010. *American Journal of Public Health.* 2015;105(2):311–6. Giesbrecht N, Huguet N, Ogden L, Kaplan MS, McFarland BH, Caetano R. Acute alcohol use among suicide decedents in 14 U.S. states: impacts of off-premise and on-premise alcohol outlet density. *Addiction*. 2015;110(2):300–7. Hemenway D, Solnick SJ. Children and unintentional firearm death. *Injury Epidemiology.* 2015;2(1). Hempstead KA, Phillips JA. Rising suicide among adults aged 40–64 years: the role of job and financial circumstances. *American Journal of Preventive Medicine*. 2015;48(5):491–500. Huguet N, Lewis-Laietmark C. Rates of homicide-followed-by-suicide among White, African American, and Hispanic men. *Public Health*. 2015;129(3):280–2. Huguet N, McFarland BH, Kaplan MS. A comparison of suicides and undetermined deaths by poisoning among women: an analysis of the National Violent Death Reporting System. *Archives of suicide research: official journal of the International Academy for Suicide Research*. 2015;19(2):190–201. Jiang Y, Perez B, Viner-Brown S. Rhode Island Violent Death Reporting System, 2004–2013. *Rhode Island Medical Journal* (2013). 2015;98(8):36–9. Kaplan MS, Huguet N, Caetano R, Giesbrecht N, Kerr W, McFarland B. Economic contraction, alcohol intoxication, and suicide: analysis of the National Violent Death Reporting System. *Injury Prevention*. 2015a;21(1):35–41. Logan JE, Skopp NA, Reger MA, Gladden M, Smolenski DJ, Floyd C. Precipitating circumstances of suicide among active duty U.S. army personnel versus U.S. civilians, 2005–2010. *Suicide and Life-Threatening Behavior*. 2015;45(1):65–77. Mezuk B, Lohman M, Leslie M, Powell V. Suicide risk in nursing homes and assisted living facilities: 2003–2011. *American Journal of Public Health*. 2015;105(7):1495–502. Schiff LB, Holland KM, Stone DM, Logan J, Marshall KJ, Martell B. Acute and chronic risk preceding suicidal crises among middle-aged men without known mental health and/or substance abuse problems: an exploratory mixed-methods analysis. *Crisis*. 2015;36(5):304–15. Sheehan CM, Rogers RG, Boardman JD. Postmortem presence of drugs and method of violent suicide. *Journal of Drug Issues*. 2015;45(3):249–62.
2016	Anestis MD. Prior suicide attempts are less common in suicide decedents who died by firearms relative to those who died by other means. *Journal of Affective Disorders*. 2016;189:106–9. Azrael D, Mukamal A, Cohen AP, Gunnell D, Barber C, Miller M. Identifying and tracking gas suicides in the U.S. using the National Violent Death Reporting System, 2005–2012. *American Journal of Preventive Medicine*. 2016;51(5 Suppl 3):S219-25. Barber C, Azrael D, Cohen A, Miller M, Thymes D, Wang DE. Homicides by police: comparing counts from the National Violent Death Reporting System, vital statistics, and supplementary homicide reports. *American Journal of Public Health*. 2016;106(5):922–7. Blair JM, Fowler KA, Betz CJ, Baumgardner JL. Occupational homicides of law enforcement officers, 2003–2013: data from the National Violent Death Reporting System. *American Journal of Preventive Medicine*. 2016;51(5 Suppl 3):S188-96. DeGue S, Fowler KA, Calkins C. Deaths due to use of lethal force by law enforcement: findings from the National Violent Death Reporting System, 17 U.S. states, 2009–2012. *American Journal of Preventive Medicine*. 2016;51(5 Suppl 3):S173-87. Jamison EC, Bol KA. Previous suicide attempt and its association with method used in a suicide death. *American Journal of Preventive Medicine*. 2016;51(5 Suppl 3):S226-33. Kaplan MS, Huguet N, Caetano R, Giesbrecht N, Kerr WC, McFarland BH. Heavy alcohol use among suicide decedents relative to a nonsuicide comparison group: gender-specific effects of economic contraction. *Alcoholism: Clinical and Experimental Research.* 2016;40(7):1501–6. Logan JE, Fowler KA, Patel NP, Holland KM. Suicide among military personnel and veterans aged 18–35 years by county-16 states. *American Journal of Preventive Medicine.* 2016;51(5 Suppl 3):S197-208. Lyons BH. Surveillance for violent deaths—National Violent Death Reporting System, 17 states, 2013. *Morbidity and Mortality Weekly Report Surveillance Summaries*. 2016;65(10):1-42. McIntosh WL. Suicide rates by occupational group—17 states, 2012. *Morbidity and Mortality Weekly Report*. 2016;65(25):641-5. Naimi TS, Xuan ZM, Cooper SE, Coleman SM, Hadland SE, Swahn MH. Alcohol involvement in homicide victimization in the United States. *Alcoholism-Clinical and Experimental Research.* 2016;40(12):2614–21. Perlis ML, Grandner MA, Brown GK, Basner M, Chakravorty S, Morales KH. Nocturnal wakefulness as a previously unrecognized risk factor for suicide. *The Journal of Clinical Psychiatry.* 2016;77(6):e726–33. Sheftall AH, Asti L, Horowitz LM, Felts A, Fontanella CA, Campo JV. Suicide in elementary school-aged children and early adolescents*. Pediatrics*. 2016;138(4). pii:e20160436. Stone DM, Holland KM, Schiff LB, McIntosh WL. Mixed methods analysis of sex differences in life stressors of middle-aged suicides. *American Journal of Preventive Medicine.* 2016;51(5 Suppl 3):S209-18. Tian N, Cui WJ, Zack M, Kobau R, Fowler KA, Hesdorffer DC. Suicide among people with epilepsy: a population-based analysis of data from the U.S. National Violent Death Reporting System, 17 states, 2003–2011. *Epilepsy & Behavior*. 2016;61:210–7.
2017	Choi NG, DiNitto DM, Marti C, Kaplan MS, Conwell Y. Suicide means among decedents aged 50+ years, 2005–2014: trends and associations with sociodemographic and precipitating factors. *The American Journal of Geriatric Psychiatry.* 2017;25(12):1404–14. Choi NG, DiNitto DM, Marti CN. Youth firearm suicide: precipitating/risk factors and gun access. *Children and Youth Services Review.* 2017;83:9–16. Choi NG, DiNitto DM, Marti CN, Conwell Y. Physical health problems as a late-life suicide precipitant: examination of coroner/medical examiner and law enforcement reports. *The Gerontologist.* 2017;59(2):356-67. Choi NG, DiNitto DM, Marti CN, Kaplan MS. Older suicide decedents: intent disclosure, mental and physical health, and suicide means. *American Journal of Preventive Medicine*. 2017;53(6):772–80. Fowler KA, Dahlberg LL, Haileyesus T, Gutierrez C, Bacon S. Childhood firearm injuries in the United States. *Pediatrics*. 2017;140(1). pii: e20163486. Frazier L, Ortega L, Patel N, Barnes J, Crosby AE, Hempstead K. Methods and findings from the National Violent Death Reporting System for identifying gang-like homicides, 2005–2008. *Journal of the National Medical Association*. 2017;109(4):272–8. Hemenway D, Solnick SJ. The epidemiology of homicide perpetration by children. *Injury Epidemiology.* 2017;4(1):5. Holland KM, Vivolo-Kantor AM, Logan JE, Leemis RW. Antecedents of suicide among youth aged 11–15: a multistate mixed methods analysis. *Journal of Youth and Adolescence.* 2017;46(7):1598–610. Kerr WC, Kaplan MS, Huguet N, Caetano R, Giesbrecht N, McFarland BH. Economic recession, alcohol, and suicide rates: comparative effects of poverty, foreclosure, and job loss. *American Journal of Preventive Medicine.* 2017;52(4):469–75. Naimi TS, Xuan ZM, Coleman SM, Lira MC, Hadland SE, Cooper SE. Alcohol policies and alcohol-involved homicide victimization in the United States. *Journal of Studies on Alcohol and Drugs*. 2017;78(5):781–8. Patton CL, McNally MR, Fremouw WJ. Military versus civilian murder-suicide. *Journal of Interpersonal Violence.* 2017;32(17):2566–90. Petrosky E, Blair JM, Betz CJ, Fowler KA, Jack SP, Lyons BH. Racial and ethnic differences in homicides of adult women and the role of intimate partner violence—United States, 2003–2014. *Morbidity and Mortality Weekly Report.* 2017;66(28):741-46. Phillips JA, Hempstead K. Differences in U.S. suicide rates by educational attainment, 2000–2014. *American Journal of Preventive Medicine*. 2017;53(4):e123-e30. Reckdenwald A, Simone S. Injury patterns for homicide followed by suicide by the relationship between victims and offenders. *Homicide Studies*. 2017;21(2):111–32. Roberts K, Miller M, Azrael D. Honor-related suicide in the United States: a study of National Violent Death Reporting System data. *Archives of Suicide Research: Official Journal of the International Academy for Suicide Research*. 2017:1–13. Stack S, Bowman B. Cultural Scripts: Analysis of suicide location in film, 1900–2013. *Sociological Focus*. 2017;50(4):346–60. Wong YJ, Wang L, Li SZ, Liu HB. Circumstances preceding the suicide of Asian Pacific Islander Americans and White Americans. *Death Studies*. 2017;41(5):311–7.
2018	Annor FB, Bayakly RA, Morrison RA, Bryan MJ, Gilbert LK, Ivey-Stephenson AZ. Suicide among persons with dementia, Georgia, 2013 to 2016. J*ournal of Geriatric Psychiatry and Neurology.* 2019;32(1):31-9. [Epub 2018 Nov 26] Annor FB, Zwald ML, Wilkinson A, Friedrichs M, Fondario A, Dunn A. Characteristics of and precipitating circumstances surrounding suicide among persons aged 10–17 Years - Utah, 2011–2015. *Morbidity and Mortality Weekly Report*. 2018;67(11):329–32. Choi NG, DiNitto DM, Marti C, Choi BY. Poisoning deaths among late-middle aged and older adults: comparison between suicides and deaths of undetermined intent. *International Psychogeriatrics*. 2018 Oct 24. [Epub ahead of print] Choi NG, DiNitto DM, Sagna AO, Marti C. Older women who died by suicide: suicide means, sociodemographic and psychiatric risk factors, and other precipitating circumstances. *International Psychogeriatrics.* 2018;30(10):1531-40 Choi NG, DiNitto DM, Sagna AO, Marti CN. Postmortem blood alcohol content among late-middle aged and older suicide decedents: associations with suicide precipitating/risk factors, means, and other drug toxicology. *Drug and Alcohol Dependence.* 2018;187:311–8. Fowler KA, Jack SPD, Lyons BH, Betz CJ, Petrosky E. Surveillance for violent deaths -National Violent Death Reporting System, 18 states, 2014. *Morbidity and Mortality Weekly Report Surveillance Summaries.* 2018;67(2):1–36. Fridel EE, Zimmerman GM. Putting homicide followed by suicide in context: do macro-environmental characteristics impact the odds of committing suicide after homicide?. *Criminology: An Interdisciplinary Journal.* 2019;57(1):34-73. [Epub 2018 Nov 29] Gollub EL, Gardner M. Firearm legislation and firearm use in female intimate partner homicide using National Violent Death Reporting System data. *Preventive Medicine.* 2018;118:216–9. Holland KM, Brown SV, Hall JE, Logan JE. Circumstances preceding homicide-suicides involving child victims: a qualitative analysis. *Journal of Interpersonal Violence.* 2018;33(3):379–401. Ivey-Stephenson AZ, Blair JM, Crosby AE. Efforts and opportunities to understand women’s mortality due to suicide and homicide using the National Violent Death Reporting System. *Journal of Women’s Health*. 2018;27(9):1073–81. Jack SPD, Petrosky E, Lyons BH, Blair JM, Ertl AM, Sheats KJ. Surveillance for violent deaths - National Violent Death Reporting System, 27 states, 2015. *Morbidity and Mortality Weekly Report*. 2018;67(11):1–32. Jiang Y, Ranney ML, Sullivan B, Hilliard D, Viner-Brown S, Alexander-Scott N. Can statewide emergency department, hospital discharge, and violent death reporting system data be used to monitor the burden of firearm-related injury and death in Rhode Island? *Journal of Public Health Management & Practice*. 2019;25(2):137-46. [Epub 2018 Jul 7] Kalesan B, Mobily ME, Vasan S, Siegel M, Galea S. The role of interpersonal conflict as a determinant of firearm-related homicide-suicides at different ages. *Journal of Interpersonal Violence.* 2018;33(15):2335–51. Kalesan B, Sampson LA, Zuo Y, Galea S. Sex and age modify the relationship between life circumstances and use of a firearm in suicide deaths across 17 U.S. states. *Journal of Affective Disorders.* 2018;236:105–11. Leavitt RA, Ertl A, Sheats K, Petrosky E, Stephenson AI, Fowler KA. Suicides among American Indian/Alaskan natives — National Violent Death Reporting System, 18 states, 2003–2014. *Morbidity and Mortality Weekly Report.* 2018;67(8):237–42. Massetti GM, Holland KM, Jack SP, Ragan KR, Lunsford NB. Circumstances of suicide among individuals with a history of cancer. *Psycho oncology*. 2018;27(7):1750-6. O’Donnell J, Logan J, Bossarte R. Ten-year trend and correlates of reported posttraumatic stress disorder among young male veteran suicide decedents-results from the National Violent Death Reporting System, 16 U.S. states, 2005–2014. *Suicide and Life-Threatening Behavior.* 2018 Nov 29. [Epub ahead of print] Petrosky E, Harpaz R, Fowler KA, Bohm MK, Helmick CG, Yuan KM. Chronic pain among suicide decedents, 2003 to 2014: findings from the National Violent Death Reporting System. *Annals of Internal Medicine*. 2018;169(7):448-55. Reckdenwald A, Szalewski A, Yohros A. Place, Injury patterns, and female-victim intimate partner homicide. *Violence Against Women.* 2018 Sep 21. [Epub ahead of print] Rockett IRH, Caine ED, Connery HS, D’Onofrio G, Gunnell DJ, Miller TR. Discerning suicide in drug intoxication deaths: paucity and primacy of suicide notes and psychiatric history. *PLoS One*. 2018;13(1):e0190200. Rockett IRH, Caine ED, Stack S, Connery HS, Nolte KB, Lilly CL. Method overtness, forensic autopsy, and the evidentiary suicide note: a multilevel National Violent Death Reporting System analysis. *PLoS One.* 2018;13(5):e0197805. Skopp NA, Holland KM, Logan JE, Alexander CL, Floyd CF. Circumstances preceding suicide in U.S. soldiers: a qualitative analysis of narrative data. *Psychological Services.* 2018 Oct 29. [Epub ahead of print] Stack S, Rockett IR. Are suicide note writers representative of all suicides? Analysis of the national violent death reporting system. *Suicide and Life-Threatening Behavior*. 2018;48(1):12–20. Stone DM, Simon TR, Fowler KA, Kegler SR, Yuan KM, Holland KM. Vital signs: trends in state suicide rates - United States, 1999–2016 and circumstances contributing to suicide-27 states, 2015. *Morbidity and Mortality Weekly Report.* 2018;67(22):617–24. Tian N, Zack M, Fowler KA, Hesdorffor DC. Suicide timing in 18 states of the United States from 2003 to 2014. *Archives of Suicide Research.* 2018 May 23. [Epub ahead of print] Williams SC, Schmaltz SP, Castro GM, Baker DW. Incidence and method of suicide in hospitals in the United States. *Joint Commission Journal on Quality and Patient Safety*. 2018;44(11):643–50. Wong YJ, Deng K, Lee CS, Grimes J, Li PFJ. Asian Pacific Islander Americans’ and White Americans’ suicide methods. *Asian American Journal of Psychology.* 2018;9(4):318–26. Yau RK, Paschall MJ. Epidemiology of asphyxiation suicides in the United States, 2005–2014. *Injury Epidemiology.* 2018;5(1):1.

^a^There were no articles published from 2002 to 2004 and there were 8 articles in 2018 that were published ahead of print

Of the 150 studies included, 104 (69.3%) studies were on suicide and 39 (26.0%) on homicide. More than three quarters of the studies (82.7%) were published in health and medical journals. Types of studies ranged from descriptive epidemiology (66.7%), risk factor analysis (20.7%), evaluation research (6.0%), trend analysis (4.7%), and data quality assessment (2.7%). Other specific topics assessed included firearms (9.3%), homicide followed by suicide (4.7%) and unintentional firearm-related deaths (2.0%). Of the 150 studies, 27 (18.0%) were based on data from single states.

**Fig. 2:**
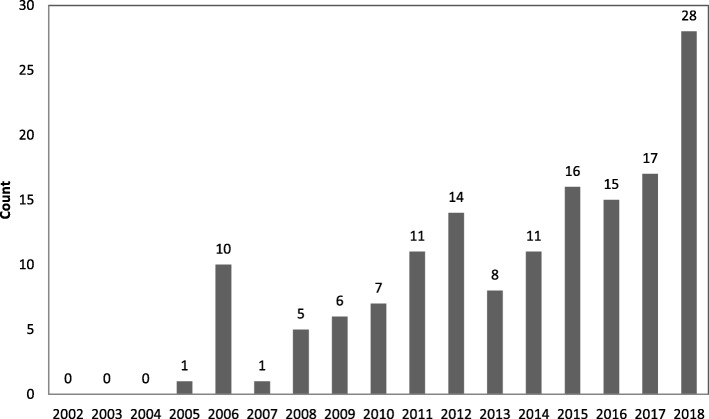
Annual frequency of published studies based on data from the National Violent Death Reporting System, 2002–2018

**Fig. 3:**
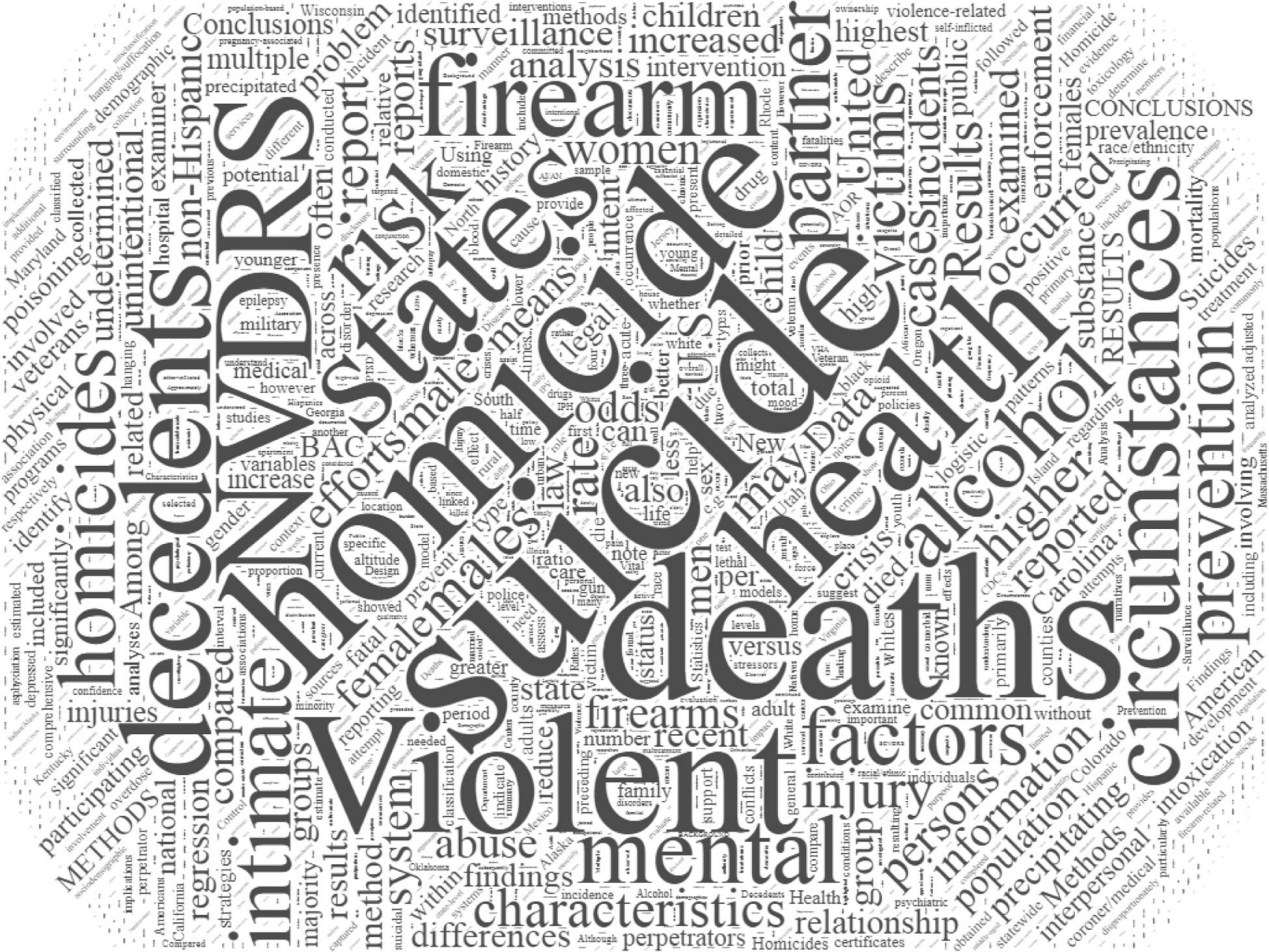
Word cloud of keywords listed in studies based on data from the National Violent Death Reporting System, 2002–2018

**Fig. 4:**
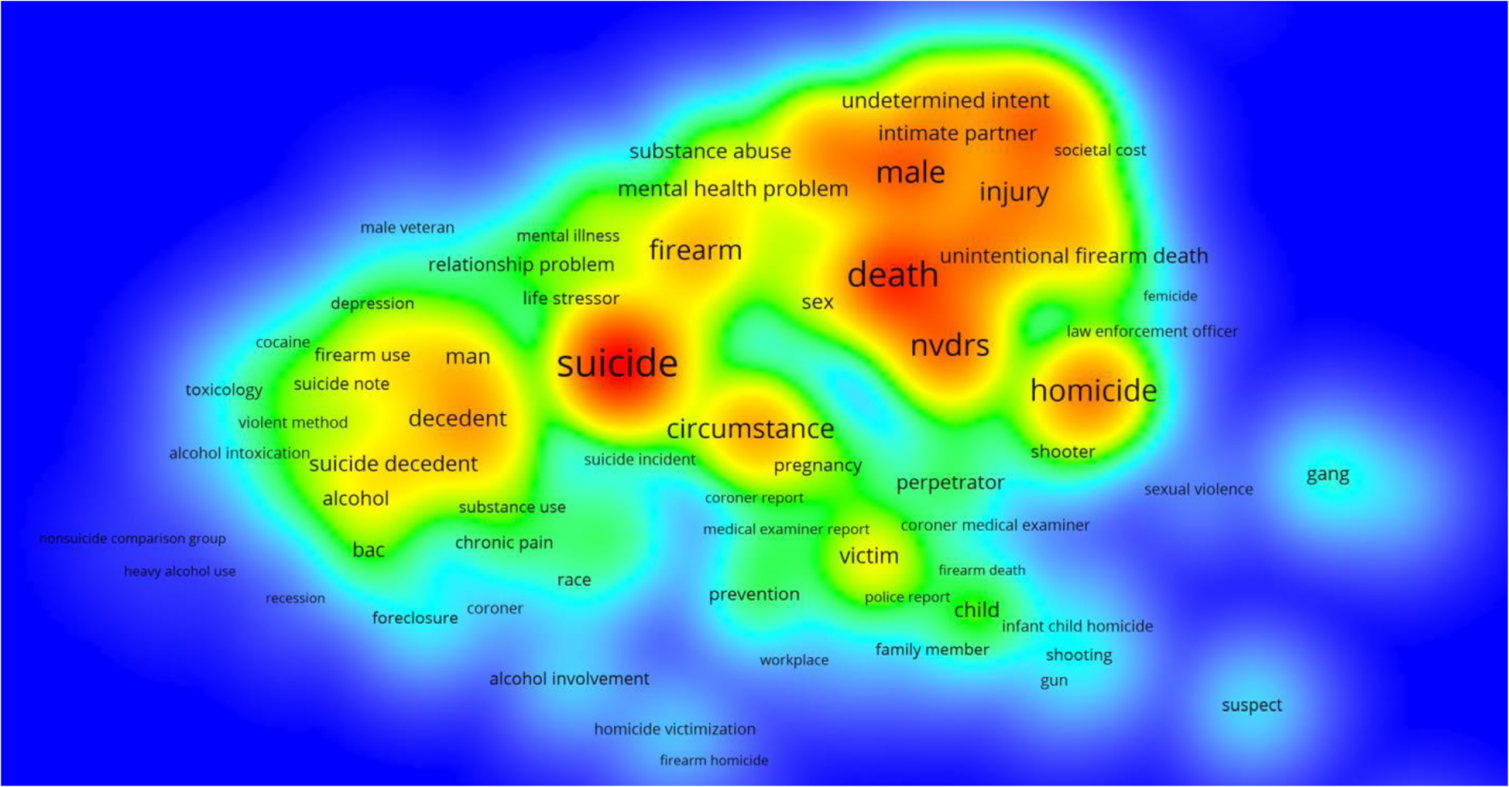
Density view of frequently used words in studies based on data from the National Violent Death Reporting System, 2002–2018

**Fig. 5:**
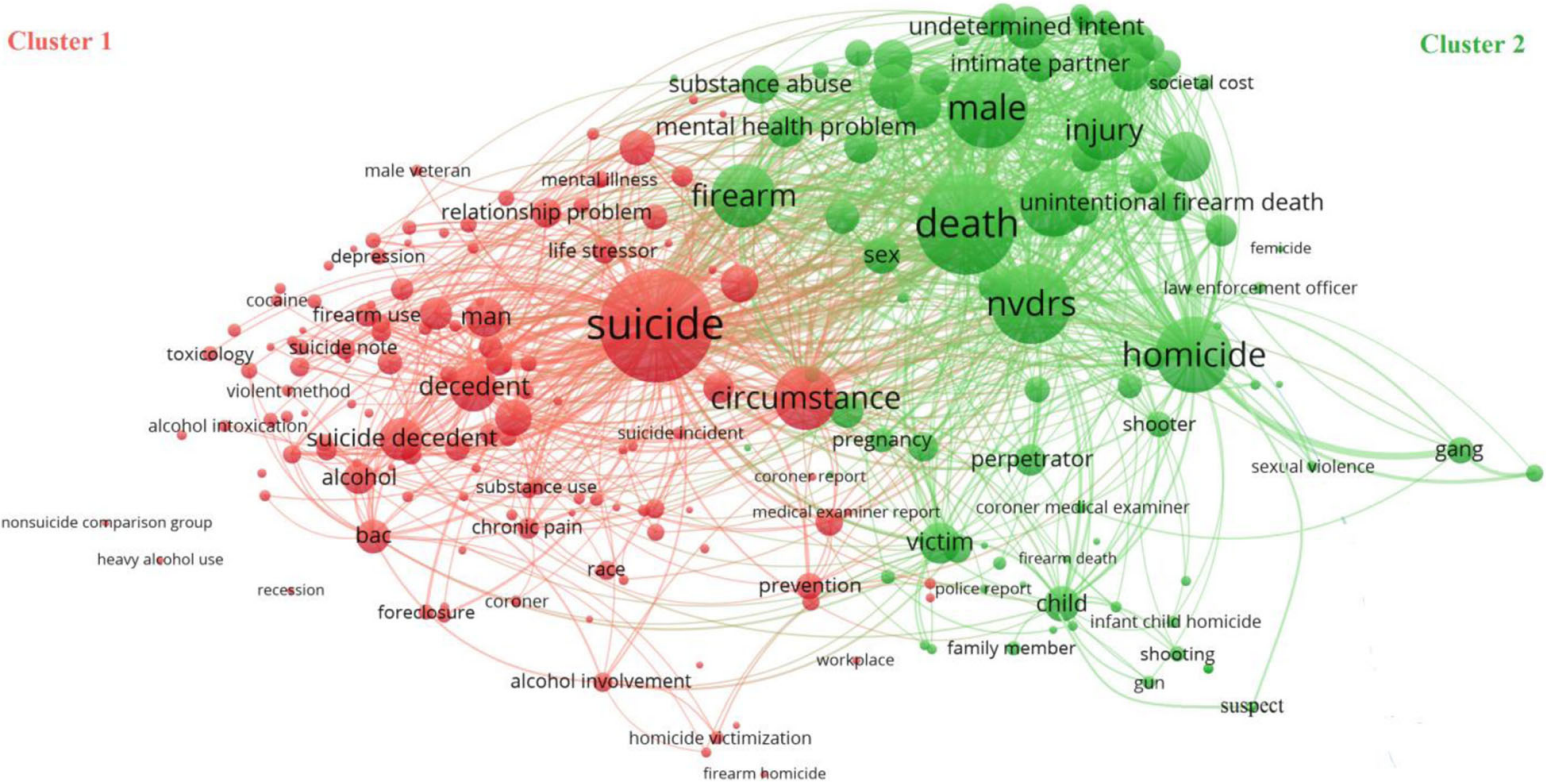
Clusters in the knowledge domains covered by studies based on data from the National Violent Death Reporting System, 2002–2018

There were no articles published between 2002 and 2004. The annual number of articles based on data from NVDRS increased from 1 in 2005 to 28 in 2018 (Fig. 2). Based on size and location of the words in the word cloud, the most frequently used words in the articles were homicide, suicide, NVRDS, and deaths (Fig. 3). Frequently used words and their interrelations are displayed in the density map (Fig. 4). Suicide was the most researched topic and was commonly linked to “circumstance,” “alcohol” and “substance abuse.” Knowledge domain mapping and network visualization revealed two major clusters and their interconnections (Fig. 5). The larger cluster was on suicide, with total links strength of 7902 and 529 occurrences (Table 2). Alcohol and substance abuse were most frequently examined risk factors associated with suicide. The second cluster was on homicide with total links strength of 3585 and 175 occurrences and was commonly linked to “firearm,” “injury,” and “gang” (Fig. 5 and Table 2).

**Table 2:** Total links strength and frequency of occurrences by major terms within each cluster in knowledge domains covered by studies based on data from the National Violent Death Reporting System, 2002–2018

Clusters	Terms	Total links strength	Occurrences
1	*Suicide*	7902	529
	*Circumstance*	2470	125
	*Alcohol*	623	37
	*Substance Abuse*	176	9
2	*Homicide*	3586	175
	*Firearm*	2547	103
	*Injury*	2236	70
	*Gang*	450	18

## Discussion

This scoping review provides a general assessment of the research utility of the NVDRS. The results indicate that research output as measured by the number of peer-reviewed publications has increased markedly in recent years. However, the majority of the authors and their publishing outlets are within the field of public health and medicine. The NVDRS is still underutilized by researchers in other disciplines, such as criminology, sociology, economics and policy. NVDRS-based studies are clustered into two knowledge domains; suicide and homicide. Knowledge domain mapping reveals that these studies covered a variety of interrelated topics. Given that suicide comprises the majority of violent deaths in the United States, it is not surprising that suicide is the most frequently researched topic covered in NVDRS-based studies. The term most commonly linked to studies on suicide is “circumstance,” followed by “alcohol,” and “substance abuse.” Homicide is the second most frequently covered topic in NVDRS-based studies. The term most commonly linked to studies on homicide is “firearm”, followed by “injury” and “gang.” Our analysis also shows that studies clustered in the two knowledge domains (i.e., suicide and homicide) are densely interlinked to overlapping networks of keywords, such as “firearm,” “mental health problem,” “substance abuse,” and “men.”

Although knowledge domain mapping sheds light on the development progress and structural relationship among NVDRS-based studies, this scoping review has several limitations. First, our search was limited to six major bibliographical databases for studies published in peer-reviewed journals. NVDRS-based studies published in the “gray” literature, such as theses, dissertations, and conference proceedings, are not included. Second, given the nature of the scoping review that focuses on general exploration of the research topic and its narrative presentation (Peterson et al. 2017), we did not evaluate the quality and findings of the individual studies. Finally, our review was restricted to assessing the research utility of the NVDRS. Use of the NVDRS for policy briefing, advocacy and other functions (Sundararaman et al. 2008) is not included in this review but could be as important as for knowledge creation.

## Conclusion

Bibliographical analysis from this scoping review indicates that research output using the NVDRS has increased substantially since 2005. With the expansion of the NVDRS, this upward trend is likely to continue in the coming years. Results from knowledge domain mapping and manual review suggest that although NVDRS-based studies have covered a wide array of topics related to suicide and homicide, they are often focused on a few putative risk factors, such as alcohol and substance abuse for suicide and firearms and gang involvement for homicide. As more data are collected and become available, the vast potential of the NVDRS for violence research and prevention will be explored by investigators from a multitude of scientific disciplines.

## Abbreviations

CDC: Centers for Disease Control and Prevention
NVDRS: National Violent Death Reporting System
NVISS: National Violent Injury Statistics System
VOS: Visualization of Similarities
